# Global Gene Expression Profiling of Myeloid Immune Cell Subsets in Response to In Vitro Challenge with Porcine Circovirus 2b

**DOI:** 10.1371/journal.pone.0091081

**Published:** 2014-03-11

**Authors:** Bettina Mavrommatis, Victoria Offord, Robert Patterson, Mick Watson, Theo Kanellos, Falko Steinbach, Sylvia Grierson, Dirk Werling

**Affiliations:** 1 The Royal Veterinary College, Hatfield, United Kingdom; 2 ARK-Genomics, The Roslin Institute & R(D)SVS, University of Edinburgh, Midlothian, Edinburgh, United Kingdom; 3 Zoetis Animal Health, Paris, France; 4 Department of Virology, Animal Health and Veterinary Laboratories Agency, Addlestone, United Kingdom; Technische Universität Dresden, Medical Faculty, Germany

## Abstract

Compelling evidence suggests that the early interaction between porcine circovirus 2 (PCV-2) and the innate immune system is the key event in the pathogenesis of Post-Weaning Multisystemic Wasting Syndrome (PMWS). Furthermore, PCV2 has been detected in bone-marrow samples, potentially enabling an easy spread and reservoir for the virus. To assess the gene-expression differences induced by an in-vitro PCV2b infection in different three different myeloid innate immune cell subsets generated from the same animal, we used the Agilent Porcine Gene Expression Microarray (V2). Alveolar macrophages (AMØs), monocyte-derived dendritic cells (MoDCs) and bone-marrow cells (BMCs) were generated from each animal, and challenged with a UK-isolate of a PCV2 genotype b-strain at a MOI of 0.5. Remarkably, analysis showed a highly distinct and cell-type dependent response to PCV2b challenge. Overall, MoDCs showed the most marked response to PCV2b challenge *in vitro* and revealed a key role for TNF in the interaction with PCV2b, whereas only few genes were affected in BMCs and AMØs. These observations were further supported by an enrichment of genes in the downstream *NF-κB Signalling* pathway as well as an up regulation of genes with pro-apoptotic functions post-challenge. PCV2b challenge increases the expression of a large number of immune-related and pro-apoptotic genes mainly in MoDC, which possibly explain the increased inflammation, granulomatous inflammation and lymphocyte depletion seen in PMWS-affected pigs.

## Introduction

PCV2, a non-enveloped, single-stranded circular DNA virus, has been recognized as the underlying agent for Post Weaning Multisystemic Wasting Syndrome (PMWS) [1], [2] and is now endemic in most pig-producing countries. Thus far, three genotypes of PCV2 have been described. PCV2a first reported in archived tissue samples, PCV2b, first reported in 2005 in North America and PCV2c recently described in Denmark [3]–[5]. Previous studies did not show differences in virulence of different PCV2 strains [1], [6]; however recent evidence suggests that mutations in circulating PCV2 strains coincided with a dramatic change of pathogenicity and clinical outcome [3], [7], with pigs carrying numerous PCV2 genotypes [8]. The fact that PCV2 has been detected in both PMWS and non-PMWS affected farms and pigs contributed to the notion that different PCV2 strains vary in their pathogenicity [9]. In that context it has to be noted that the dominant PCV2 strain circulating on severely-affected farms in the UK was grouped into genotype PCV2b, as determined in a recent cross-sectional study involving 147 pig farms across England [10].

Whereas the majority of endemic diseases can be controlled by vaccination or eradication of the pathogen, these approaches are less successful in the control of multi-factorial diseases. PMWS represents a typical multi-factorial disease, with pigs between 5 and 12 weeks of age being affected, and which is characterized by increased mortality, weight loss, wasting, dyspnoea and enlarged lymph nodes [8], [11], [12]. Indeed, vaccination against PCV2 seems to be successful in that it is reducing losses on affected farms, but is not inducing sterile immunity and vaccinated pigs still seem to harbour and potentially shed PCV2 [13], [14]. In this respect, the infected cells are of great importance for the distribution of PCV2.

Viruses have evolved complex immune evasion strategies for protection against host immune responses [15], and PCV2-infected pigs seem to harbour the virus in different immune cell subsets [13], [14] without infected cells showing signs of functional differences. Particularly alveolar macrophages (AMØs) and other cell-types of the monocyte/macrophage lineage seem to act as reservoirs and Trojan horse for PCV2, subsequently infecting other immune cells and specifically bone-marrow cells [14], [16]. Lymphocyte depletion, secondary infections with opportunistic pathogens, induction of apoptosis and other changes in immune cell subpopulations and PBMCs are all common characteristics of PMWS in severely affected pigs, strongly suggesting an immunosuppressive status [13], [17], [18]. Indeed, PCV2b-infected plasmacytoid dendritic cells (pDC) were shown to be unresponsive to exposure to further “danger-signals” [19], [20], supporting the notion that PCV2 induces a status of immunsuppression. However, the mechanism by which the immune system is altered remains unclear. A proposed mechanism suggests the presence of immunomodulatory CpG motif in the small circular genome of PCV2 inhibiting critical cytokine secretion [21], [22].

In the last decade, gene expression profiling microarrays have been widely used to reveal the effects of pathogens on host cells and tissues aiming to gain insight into the molecular mechanisms involved in the host-pathogen interactions [23], [24], and a limited number of studies have been performed applying the benefits of microarrays to elucidate the pathogenesis of PCV infection [24]–[27]. In all of these studies, tissues from PCV-infected pigs, rather than specific cellular subsets were analysed. Whereas this allows for a discrimination of genes being differentially expressed in different tissues as a result of the infection, it does not allow for the discrimination of PCV-induced effects in specific cell-types. These *ex vivo* data showed differential expression of numerous genes in PCV2-infected animals involved in innate immune defence (TLR1, CD14 and CD180), immunosuppressive responses (FGL2 and GPNMB) and pro-inflammatory signals (galectin-3) [24].

Due to the difficulty of reproducing an experimental model of PMWS and lack of advanced pig molecular markers [28], the objective of the present study was to analyse the early molecular mechanisms involved in PCV2b infection of three defined immune cell subsets generated from the same animals, using a genome-wide expression approach. Information obtained from this study will increase our understanding of the features of PCV2 infection during the onset of immune responses *in vivo*.

## Materials and Methods

### Ethics Statement

All animal studies were performed according to the regulations and guidance provided under the UK Home Office Animals (Scientific Procedures) Act 1986. Experimental protocols were approved under project licence number PPL 70/7219, as well as the RVC Ethics and Welfare Committee.

### Animals

Six crossbred Large White×Landrace pigs (*Sus scrofa*), free of PCV1, PCV2, swine influenza strains H1N1, H1N3, and H1N5, PRRSV and *Mycoplasma hyopneumoniae*, as confirmed by qPCR and antibody testing, were sourced from the Animal Health and Veterinary Laboratories Agency (AHVLA) and housed at the pig unit of the Royal Veterinary College. At 6 months of age, all six pigs were humanly euthanized. During housing, PCV2 status of the pigs was repeatedly confirmed by ELISA (antibody) and quantitative real-time PCR on serum. All six pigs remained PCV2 negative throughout.

### Generation of immune cell subsets

From each individual animal, the following immune cell subsets were generated:

#### Porcine alveolar macrophages (AMØs)

Porcine AMØs were obtained from the lungs of each euthanized PCV2-negative pig (n = 6). Briefly, approximately 2×500 ml of sterile PBS (containing 200 µg ml^−1^ gentamycin and 2.5 µg ml^−1^ amphotericin B) were delivered into the freshly isolated lungs via tracheal intubation. Following gentle manipulation to detach loosely adherent cells, the aspirate was removed and the resulting cell suspension was placed in a sterile 500 ml bottle. AMØ were recovered following centrifugation (200× *g* for 10 min at 4°C), washed once and resuspended in growth medium (RPMI 1640 supplemented with 10% (v/v) porcine serum, 200 µg ml^−1^ Pen/Strep and 0.5 µg ml^−1^ amphotericin B). AMØs were seeded at 1×10^6^ cells ml^−1^ and incubated at 37°C/5% CO_2_ in 6-well plates (Nalgene, USA). The majority of isolated cells expressed the surface antigens CD14 and MHC class II, as analysed by flow-cytometry (data not shown).

#### Generation of monocyte-derived dendritic cells (MoDCs)

Just before euthanasia, 500 ml of blood was collected from each pig ( = 6) into sterile bottles, containing acid citrate dextrose solution (0.22 M D(+)glucose, 0.2 M sodium citrate and 0.14 M citric acid) at 10% of the total volume. The blood was divided into 50 mL falcon tubes and centrifuged at 1000× *g* for 25 min. The buffy coat was removed and collected in a sterile glass bottle, diluted 1∶2 (v/v) with calcium/magnesium free PBS (PBSa) at room temperature. Peripheral blood mononuclear cells (PBMCs) were isolated from the buffy coat via density centrifugation over Histopaque (Histopaque 1.077 g ml^−1^, Sigma, Poole, Dorset, UK) as described previously [29]. After centrifugation (800× *g* for 25 min at 22°C), the mononuclear cells were recovered from the interface formed between the cell suspension and the Histopaque and washed twice by dilution in cold (4°C) PBS-A 0.03% (w/v) EDTA (PBS/EDTA) and centrifugation (350× *g*/10 min/4°C). CD14^+^ monocytes were isolated by positive selection using a MACS system (Miltenyi Biotech, Bergisch Gladbach, Germany), according to the manufacturer's protocol. The cells were cultured for 7 days in DMEM medium with porcine serum (10% v/v), recombinant porcine (rp) granulocyte–macrophage colony-stimulating factor (GM-CSF, 150 ng ml^−1^) and rp interleukin-4 (IL-4, 100 U ml^−1^). RpIL-4 and rpGM-CSF were prepared in house, and bioactivity of both cytokines was determined using a TF-1 cell bioassay, with the IL-4 concentration giving half-maximum proliferation being defined as 1 unit. Resulting cells were CD14^low^, and expressed the DC-SIGN molecule, consistent with the phenotype reported by Huang *et al.*, 2009 [30].

#### Generation of bone marrow cells (BMCs)

BMCs were isolated from the sternum of each pig (n = 6) as previously described [31]. Briefly, the sternum was opened and flushed with PBS/0·03% EDTA (w/v) at 37°C. The obtained cell suspension was depleted of erythrocytes and mature granulocytes by centrifugation over Histopaque (1.077 g ml^−1^, Sigma, Poole, Dorset, UK) at 1000× *g* for 40 min at room temperature. After a further two PBS/EDTA washes (250× *g*/10 min/4°C), the cells were cultured for 7 days in αMEM medium with porcine serum (10% v/v), 200 µg ml^−1^ Pen/Strep, and 0.5 µg ml^−1^ amphotericin B.

#### Virus preparation for infection

To avoid potential false-positive results due to LPS contaminations of reagents/media, all substances used were tested for their LPS content using the Endosafe-PTS system (Charles River, Charleston, USA). Samples with an endotoxin content below 0.01 EU ml^−1^ were considered as LPS-free.

A previously characterized PCV2b isolate from the UK (GenBank accession number JX193799; [10]) was used to generate the virus stock for the experimental infections. The virus was propagated in type-I IFN^KO^ PK15-ALR-NPro cells, free of PCV1 and PCV2. Cells were cultured in MEM containing Earle's salts supplemented with 10% (v/v) tetracycline-free foetal bovine serum (FBS) (Clontech, Saint-Germain-en-Laye, France). To induce the tetracycline-regulated expression of the IFN-KO, tetracycline was added 2 h pre-inoculation. Cell monolayers were inoculated at 50% confluence. After 18 h incubation at 37°C, the inoculum was removed and retained, and the monolayer treated with 300 mM D-glucosamine in Hanks balanced salt solution (HBSS) for 30 min at 37°C. After removal of the glucosamine and subsequent washes with HBSS, cultures were overlaid with retained inoculum. Cultures were overlaid with media 24 h post-inoculation. After a further 3 days incubation media was removed and retained and virus harvested from trypsinised cells by freeze-thawing. The cell lysate was clarified by centrifugation at 1,000× g for 5 min at 4°C and the supernatant added to the retained media. This virus suspension was then concentrated approximately 10-fold using dialysis tubing (Spectra/Por, Biotech Cellulose Ester membrane; Spectrum Europe B.V.) in polyethylene glycol (PEG 12000 flake; Whyte Chemicals Ltd.) at 4°C. Concentrated virus suspension was subsequently dialysed in MEM overnight and aliquoted. PCV2 stocks were titrated on PK15-ALR-NPro cells as described elsewhere [1]. The titre of the virus stock was determined as 10^6.05^ TCID_50_ ml^−1^.

In line with the general accepted rules for work with viruses, and similar as described by others [14], [20], [32], supernatant of type-I IFN^KO^ PK15-ALR-NPro cells was used as true mock-infection control to counteract gene expression changes induced by the cell culture supernatant of PK15-ALR-NPro cells rather than being a true result of PCV2b infection.

All three cell types generated from each pig were exposed on the same day to either PCV2b at an MOI of 0.5 or remained uninfected in six-well-plates for 0 h, 1 h and 24 h at 37°C. At the 0 h time-point, cells were overlaid with PCV2b stock at an MOI of 0.5 and immediately washed and lysed; this served as a further control for normal gene expression within the cells. At time-points indicated, cells were lysed in 600 µL GTC buffer (4 M guanidine thiocyanate, 25 mM sodium citrate, 0.5% (w/v) sodium N- lauroylsarcosine, 0.1 M 2-mercaptoethanol, pH 7) after two washes, as required for subsequent RNA extraction.

### Assessment of infection by qPCR

The number of PCV2 copies per ng of cDNA isolated from each cell type was determined by comparison to known standards using qPCR. Each sample was measured in triplicate and in a final volume of 20 µl per well in Microamp fast optical 48-microtiter well plates (Applied Biosystems). Each well contained, 2 µl of cDNA (standard or test sample), 10 µl of 2× TaqMan Universal Master Mix II (Applied Biosystems), 50 pmol of each primer (Forward: 5′-GCTCTYTATCGGAGGATTAC-3′, Reverse: 5′-ATAAAAACCATTACGAWGTGATA-3′) (MWG) and 2.5 µM of TaqMan probe (5′FAM-CCATGCCCTGAATTTCCATATGAAAT-3′TAMRA) (Applied Biosystems). The volume of each well was adjusted to 20 µl by addition of nuclease-free water (Sigma-Aldrich). The TaqMan probe and the primers were designed to target a partial (137 bp) sequence of the ORF2 of PCV2 [33]. Standard measurements were performed in a 10-fold serial dilution from 10^9^ PCV2 copies to 0 copies. After plate set up, the qPCR was performed in a StepOne Real-time PCR machine (Applied Biosystems) and StepOne software version 2.2.2 (Applied Biosystems). The cycling conditions were 95°C for 10 mins for polymerase activation followed by 40 cycles of denaturation at 95°C for 15 secs and annealing/extension at 55°C for 1 min. Data was analysed using either StepOne software version 2.2.2 (Applied Biosystems) or Excel 2010 (Microsoft). Ct values of triplicate sample and standard measurements were averaged and this average Ct value was used to calculate the PCV2 ORF2 copy number in samples by comparison to the known standards. The copy number in each sample was then divided by the nanograms of DNA in that sample to give the PCV2 copy number per ng of DNA.

#### Microarray hybridizations and data analysis

From all cell types generated from each pig, total RNA was extracted at each time point indicated from cells either mock- or PCV2-infected, using the RNeasy kit (Qiagen, Crawley, UK) according to the manufacturer's instructions, including an RNase-free DNase I (Qiagen, Crawley, UK) digestion step at the time-points indicated. RNA integrity and quality was determined using the Agilent 2100 Bioanalyzer (Agilent Technologies, Wokingham, UK). Up to 50 ng of total RNA with RIN values ≥8 was reverse transcribed to cDNA and then transcribed into Cy3-labelled cRNA using the Low Input Quick Amp Gene Expression Labeling Kit (Agilent, Santa Clara, CA) according to the manufacturer's instructions. Hybridization to the Agilent 4×44K Porcine Gene Expression Microarray (V2; Agilent) was performed on a Tecan Hybridization Station HSPro400 (Tecan, Maennersdorf, Switzerland). Microarrays were scanned with an Agilent C Scanner (Agilent Technologies, Wokingham, UK).

Following quality control checks and principal component analysis (PCA), a subset of 12 arrays were removed. A total of 100 arrays were analysed with 38, 21 and 41 arrays representing AMØs, BMCs and MoDCs respectively. Pre-processing steps of background correction and between-array normalization were performed prior to analysis using the software packages R (http://www.r-project.org) and Bioconductor (http://www.bioconductor.org) [34]. Differential expression analysis was performed by the Bioconductor package *limma* using the functions *lmFit* and *eBayes*. With increasing advancements in microarray technology, numerous studies have now used a −1.5≤ fold change (FC) ≥1.5 [35], [36] and have verified the biological significance of these transcripts [37], [38], consequently, in this study differences in gene expression between the treatment groups were considered significant using false discovery rate (FDR)-adjusted *P*<0.05 [40] and a −1.5≤FC≥1.5 as a cut-off.

#### Systems Biology Analysis

For further analysis, all differentially expressed genes (*P*<0.05) were imported into the Ingenuity Systems Pathway Analysis program (IPA; Ingenuity Systems, Redwood City, CA, USA; http://www.ingenuity.com). Canonical pathways analysis identified the pathways from the IPA library of canonical pathways that were most significant to the data set. Transcripts from the data set that met the −1.5≤FC≥1.5 and a FDR-adjusted *P*<0.05 cut-off and were associated with a canonical pathway in the Ingenuity Knowledge Base were considered for the analysis. The significance of the association between the data set and the canonical pathway is measured by considering: (1) the number of focus genes that participate in that process, (2) the total number of genes that are known to be associated with that process in the selected reference set and lastly, Fischer's exact test is used to calculate a p-value and the corrected p-value, using the Benjamini-Hochberg method [39], is displayed for the Functions and Canonical Pathways (IPA).

#### Real time quantitative reverse transcription PCR (qRT-PCR) analysis

The significance of differential expression between PCV2b- challenged and unchallenged cells uncovered by Agilent microarray analyses was further validated using real-time PCR. cDNA was prepared from total RNA isolated from each sample analysed in the microarray study using a High Capacity cDNA Reverse Transcription Kit (Applied Biosystems, Life Technologies Corporation, Warrington, UK). cDNA conversions were performed in 20 µl reaction volumes using random primers as per manufacturer's instructions.

Taqman gene expression assays (Applied Biosystems, Life Technologies Corporation, Warrington, UK) were used to determine expression of *CXCL2*, *IL-8*, *IL-1β*, *IL-1α*, *TNF*, and *PTGES* in cDNA samples prepared above according to the manufacturer's instructions. After evaluation of several house-keeping genes according to the MIQE-guidelines [40], glyceraldehyde-3-phosphate dehydrogenase (GAPDH) was chosen as the most stable one with low variation between samples in the microarray analysis. qPCR was carried out in 48-well optical plates (Applied Biosystems, Life Technologies Corporation, Warrington, UK) and each sample was measured in triplicate. Measurements were made using a StepOne qPCR machine (Applied Biosystems, Life Technologies Corporation, Warrington, UK) and StepOne software version 2.1 (Applied Biosystems, Life Technologies Corporation, Warrington, UK). PCR thermal cycling conditions for each amplicon comprised of one cycle at 95°C for 10 mins, followed by 40 cycles at 95°C for 15 seconds and 60°C for 60 seconds.

Changes in gene expression revealed by qPCR were calculated by the 2^ΔΔ^Ct method [41]. Normalization was carried out by dividing their relative expression level by the relative expression level of GAPDH.

## Results

### Confirmation of PCV2b infection in all cell types over time

**Figure 1:**
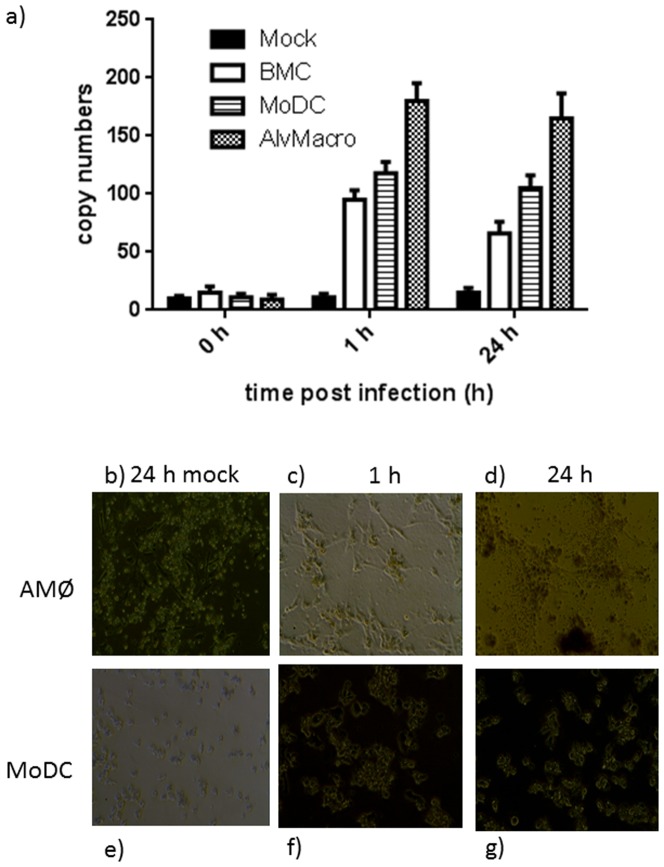
Quantitative PCR of PCV2b copy-numbers in immune cell subsets. Cells were prepared and infected as described, and PCV2 copy numbers analysed at the time-points indicated (Fig. 1a). Effect of PCV2 infection on immune cell subsets was also investigated by light microscopical examination of infected cells (AMØs Fig. 1 c and d: MoDC Fig. 1 f and g) compared to mock-infected cells (Fig. 1 b and e, respectively). Magnification ×40.

Quantitative PCR revealed the presence of increasing copy-numbers of PCV2b in all three cell-types, with the highest copy number being detected in alveolar macrophages (Fig. 1a). In addition, specifically AMØs showed substantial cell-morphological changes between 24 h mock-infected (Fig. 1b) and 24 h after infection (Fig. 1d), as well as between 1 h after infection (Fig. 1c) and 24 h after infection (Fig. 1d), as seen by light-microscopy, compared to MoDC infected for the same length of time (Fig. 1 e–f).

### PCV2b challenge induces differential gene expression in all three immune cell subsets, with monocyte-derived dendritic cells showing highest differences

Differences in cell-specific gene expression of PCV2b challenged and unchallenged immune cell subsets derived from individual animals (MoDCs, AMØs and BMCs) were assessed using the Agilent Porcine Gene Expression Microarray with a cut-off p-value of p<0.05 and a fold range of −1.5≤FC≥1.5.

Interestingly, some genes were differentially regulated at timepoint 0. We hypothesise at the moment that this is not due to the PCV2 infection, rather than handling of cells (adding virus, washing of cells).

**Figure 2:**
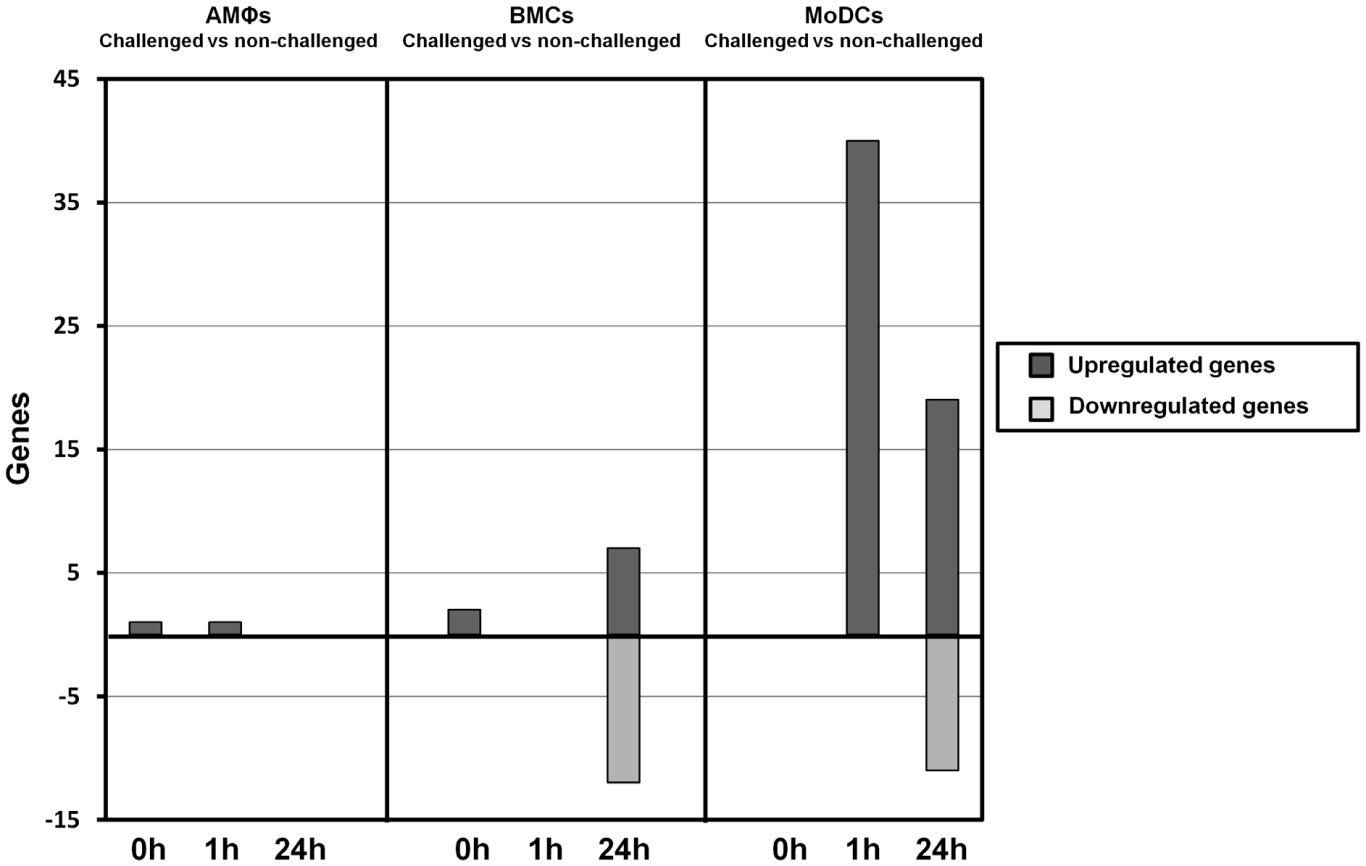
Differentially expressed genes in PCV2b-challenged and control immune cell subsets. Differentially expressed genes at each time-point are shown for the two treatment comparisons (*P*<0.05, −1.5≤ fold change ≥1.5, N = 6).

**Figure 3:**
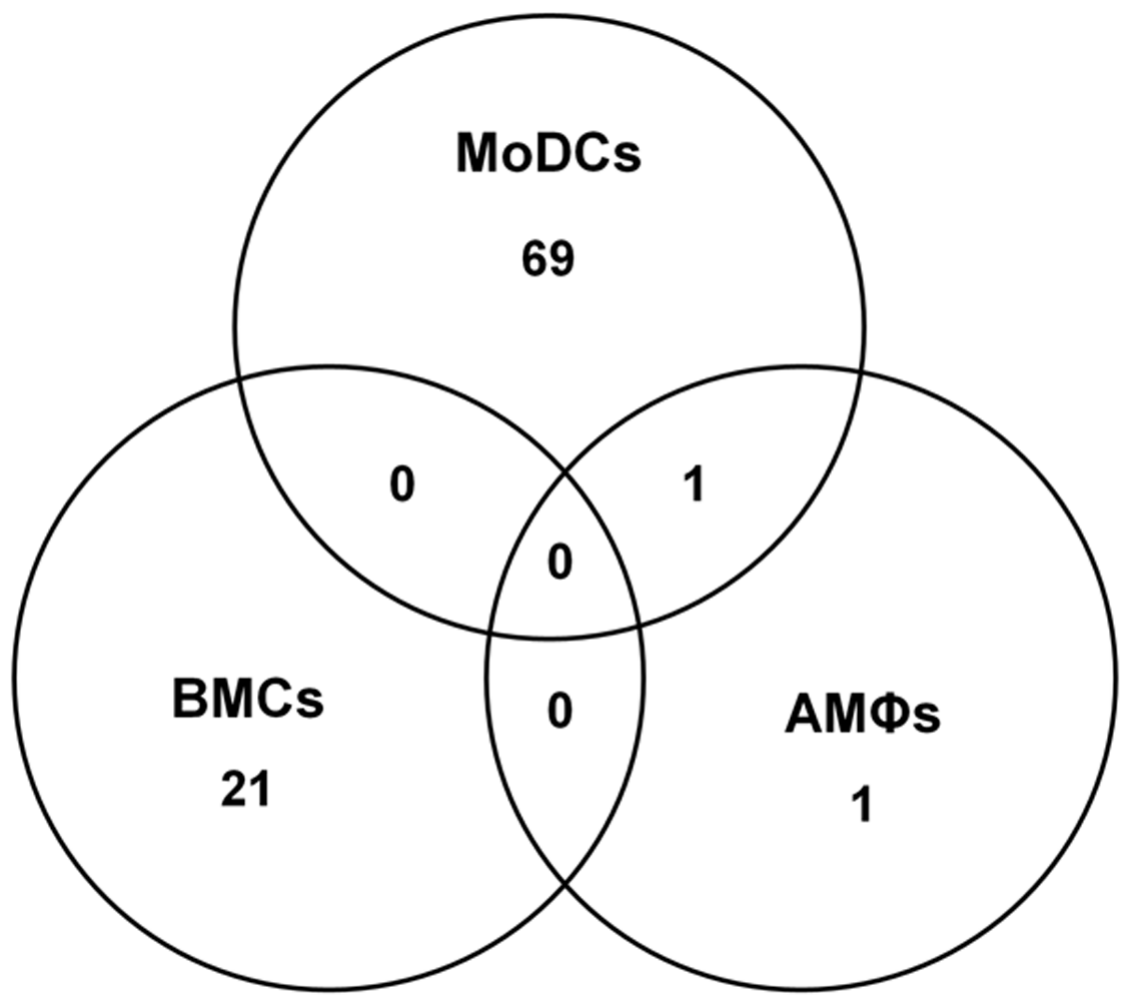
Transcriptome differences between immune cell subsets challenged with PCV2b. Venn diagram of differentially expressed genes after challenge with PCV2b is shown for each immune cell subset (P<0.05, −1.5≤ fold change ≥1.5).

Overall, 92 genes showed differential expression, with the number of differentially expressed genes increasing as infection progresses (Fig. 2). As indicated above, all three immune cell subsets were responsive to PCV2b infection and showed differential gene expression at an early (1 h) and late (24 h) time-point. Interestingly however, substantial differences were seen between the response of the different cell subsets to PCV2b exposure, resulting in a minimal overlap of differentially-expressed transcripts between MoDCs, AMØs and BMCs (Fig. 3). Among the immune cell subset studied, and compared to mock-infection using the cell culture supernatant of PK15 cells as control, MoDCs showed the most marked response to PCV2b challenge with 40 and 30 transcripts differentially-regulated at 1 h and 24 h, respectively (Fig. 2), whereas AMØs hardly reacted to the infection at all, and BMCs showed the greatest number of down-regulated genes (Fig. 2).

Independent of the cell-type, down-regulation of transcripts appeared only 24 h post PCV2b-challenge, at a time-point when PCV2b replication seemed to drop (see Fig. 1). At the early time point (1 h p.i.), the profiles of gene expression in un-challenged and PCV2b-challenged AMØs and BMCs were very similar, with only one transcript (*TNF*) was significantly altered in AMØs (Tab. 1). PCV2b challenge, however, had a clear early effect on MoDCs, which showed 40 differentially regulated transcripts (Tab. 1, Fig. 3, Tab. S1, Tab. S2), all of which were upregulated. These transcripts included a plethora of genes involved in inflammation and inflammatory responses, including NF-κB-inducible chemokine and cytokine genes such as the (C-C motif) ligand 20 gene (*CCL20*), interleukin 1 alpha and beta genes (*IL-1α* and *IL-1β*), chemokine (C-X-C motif) ligand 2 (*CXCL2*), other molecules involved in immune cell trafficking (*ADM, CCL4, MAP3K8, NFKBIA, OSM, STAB1, TIMP1* and *TNF*), antiviral response genes (*IRF1*), and apoptosis-related genes (*HBEGF, IRF1 (includes EG:16362), IER3, JAG1* and *TNFAIP3*).

**Table 1:** Annotated transcripts differentially expressed in AMØs, MoDCs and BMCs at 0 h, 1 h and 24 h p.i. following PCV2b infection.

*Cell type*	*Time-point p.i.*	*Gene symbol*	*FC*	*Gene description*
**AMØs**	**0 h**	*PRDX6*	2.58	Peroxiredoxin-6
	**1 h**	*TNF*	2.32	Tumor necrosis factor
**MoDCs**	**1 h**	*IL1B*	6.47	Interleukin 1-beta
		*CXCL2*	4.66	Chemokine (C-X-C motif) ligand 2
		*IL8*	4.4	Interleukin 8
		*TNF*	4.17	Tumor necrosis factor
		*IER3*	3.4	immediate early response 3
		*CCL4*	3.37	Chemokine (C-C motif) ligand 4
		*ADM*	3.27	adrenomedullin
		*IL1A*	3.2	Interleukin-1 alpha
		*MAP3K8*	2.91	Mitogen-activated protein kinase 8
		*BTG2*	2.35	B-cell translocation gene 2
		*RND3*	2.29	rho Family GTPase 3
		*JAG1*	2.15	jagged 1
		*TNFAIP3*	2.14	tumor necrosis factor, alpha-induced protein 3
		*IRF1*	2.1	interferon regulatory factor 1
		*NFKBIA*	2.08	NF-kappa-B inhibitor alpha
		*IRG1*	2.01	immune-responsive gene 1 protein homolog
		*MFSD2A*	1.98	Major facilitator superfamily domain-containing protein 2
		*OSM*	1.86	oncostatin M
		*HBEGF*	1.84	heparin-binding EGF-like growth factor
		*CCL20*	1.82	chemokine (C-C motif) ligand 20
		*STAB1*	1.82	stabilin 1
		*TOB1*	1.73	transducer of ERBB2, 1
		*SLC2A6*	1.71	solute carrier family 2 (facilitated glucose transporter), member 6
		*CSRNP1*	1.68	cysteine-serine-rich nuclear protein 1
**MoDCs**	**24 h**	*VDAC1P5*	4.06	voltage-dependent anion channel 1 pseudogene 5
		*S100A9*	3.3	S100 calcium binding protein A9
		*COX3*	3.21	cytochrome oxidase 3
		*ARHGAP25*	2.85	Rho GTPase activating protein 25
		*SLC25A6*	2.71	solute carrier family 25 (mitochondrial carrier; adenine nucleotide translocator), member 6
		*RPL32*	2.46	ribosomal protein L32
		*MRPL18*	2.28	mitochondrial ribosomal protein L18
		*RTN4*	2.25	reticulon 4
		*TGH2*	2.17	tissue transglutaminase homologue
		*PDLIM1*	2.1	PDZ and LIM domain 1
		*PLCXD1*	1.99	phosphatidylinositol-specific phospholipase C, X domain containing 1
		*CASP10*	1.78	caspase 10, apoptosis-related cysteine peptidase
		*RANBP1*	1.77	RAN binding protein 1
		*VDAC2*	1.66	voltage-dependent anion channel 2
		*FCN1*	−1.75	ficolin (collagen/fibrinogen domain containing) 1
		*BTC*	−1.77	betacellulin
		*ITGB5*	−2.03	integrin, beta 5
		*PIK3IP1*	−2.05	phosphoinositide-3-kinase interacting protein 1
		*SEPP2*	−2.08	septin 2
		*RNASE4*	−2.27	ribonuclease, RNase A family, 4
		*DKK3*	−2.29	dickkopf 3 homolog
		*DUOXA2*	−3	dual oxidase maturation factor 2
**BMCs**	**0 h**	*PLDN*	1.56	pallidin homolog
**BMCs**	**24 h**	*Es25*	3.9	Esterase 25
		*IGSF8*	2.78	immunoglobulin superfamily, member 8
		*PTGES*	2.54	prostaglandin E synthase
		*TMEM237*	−1.6	transmembrane protein 237
		*MMRN2*	−1.72	multimerin 2
		*MYL1*	−2.27	myosin, light chain 1
		*PEG10*	−2.57	paternally expressed 10

A total of 93 transcripts showed differential expression. Annotated transcripts are listed in each immune cell subset from the highest to the lowest fold-change at the different time points p.i.

At the late time-point, comparison of PCV2b-challenged immune cell subsets and non-challenged control cells revealed a total of 49 differentially expressed genes. Among the 30 genes identified in MoDCs, 19 showed upregulation whereas 11 genes were down-regulated (Tab. 1, Fig. 2, Tab. S2). The main transcripts with increased expression in MoDCs were *VDAC1P5* (also known as VDAC5P or VDAC3, a voltage-dependent anion-selective channel protein) the myeloid-related protein *S100A9* (potent chemotactic factor for immune cells) and *COX3* (involved in prostaglandin biosynthesis). Other upregulated transcripts included genes with apoptotic function (*CASP10*, *RANBP1*, *VDAC2* and *SLC25A6*). Down-regulated transcripts with a FC≤−1.5 included *ITGB5*, *PIK3IP1*, *SEPP2*, RNASE4, *DKK3* and *DUOXA2*.

Only seven genes were significantly upregulated in BMCs, whereas 12 transcripts were down-regulated. Two of the top-ranking genes (ID *BW961486* and *13183*) could not be annotated, however, *IGSF8*, a member of the immunoglobulin protein superfamily functioning in cell migration and viral infection, and *PTGES*, an inflammatory mediator, showed upregulation in BMCs 24 h after PCV2b-challenge (Tab. 1, Tab. S3). Four of the 12 transcripts downregulated in PCV2b-challenged BMCs were annotated and included the transmembrane protein 237 (*TMEM237), MMRN2, MYL1* and *PEG10*, a paternally-expressed negative regulator of TGFβ receptor signalling (Tab. 1, Tab. S3).

To corroborate these findings, quantitative real-time PCR analysis was performed for several key pro-inflammatory mediators, these included *CXCL2*, *IL-8*, *IL-1β*, *IL-1α*, *TNF* and *PTGNES* (Tab. 2). In general, the direction of upregulation correlated well between the two platforms; however, the magnitude of fold change was generally higher in the quantitative PCR approach.

**Table 2:** Validation of microarray data by qRT-PCR.

*Gene name*	*TaqMan® Gene Expression Assay*	*Microarray FC*	*qRT qPCR FC*
*CXCL2*	Ss03378360_u1	4.66	10.22
*IL-8*	Ss03392437_m1	4.40	17.58
*IL-1β*	Ss03393804_m1	6.47	191.21
*IL-1α*	Ss03391335_m1	3.20	18.09
*TNF*	Ss03391318_g1	4.17	8.32
*PTGNES*	Ss03392129_m1	2.54	11.92

### PCV2b infection in MoDC promotes upregulation of genes with granulocyte infiltration and apoptotic function

Ingenuity Systems Pathway Analysis (IPA) allowed the identification of canonical pathways and functional processes affected by PCV2b challenge. Pathways that were differentially affected between PCV2-b challenged and non-challenged MoDCS at 1 h p.i. primarily involved the inflammatory responses and related functional groups including *NF-κB Signalling* (1^st^ ranked; *P = 3.61E-09*) with seven out of the 175 genes present in this pathway significantly affected, *Role of proinflammatory hypercytokinemia/hyperchemokinemia* (associated with Influenza pathology; Suppl. Fig. 1), *Innate and Adaptive Immune Cell Communication* (3^rd^ ranked), *PPAR-*, *IL-6-* (4^th^, 5^th^ respectively) and *Communication between Innate and Adaptive Immune Cells* (*P = 2.25E-07*) (Tabl. S1, Tab. S4).

**Figure 4:**
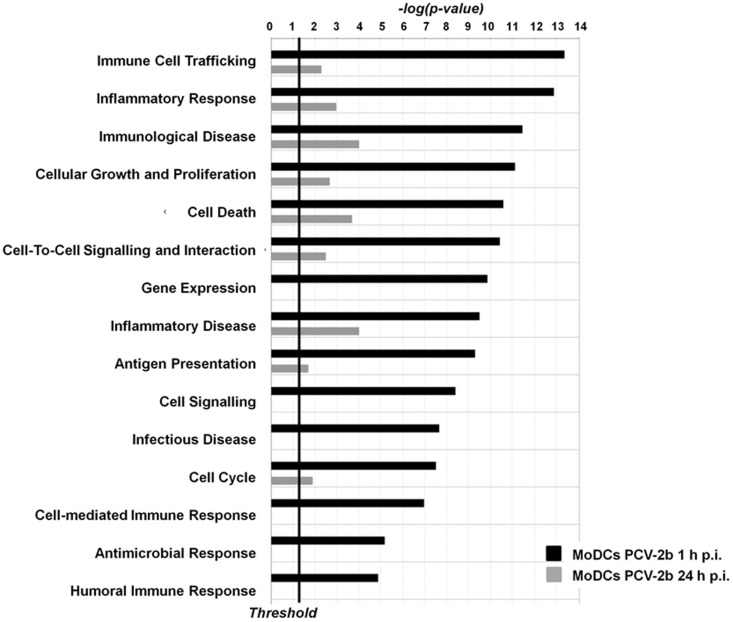
Biological process analysis of genes differentially expressed in MoDCs at 1 h and 24 h post PCV2b challenge. Differentially expressed genes (P<0.05) were imported into IPA Ingenuity software to determine significantly enriched biological processes. Data represent the distribution in cell function categories of statistically significantly enriched biological processes (P<0.05) at 0 h and 24 h post PCV2b challenge.

Significant upregulation of five genes within *proinflammatory hypercytokinemia/hyperchemokinemia* pathway (Fig. S1) indicated a strong pro-inflammatory response associated with PCV2b challenge in MoDCs leading to the recruitment of immune cells to the site of infection. As unique histopathological characteristic of PMWS-affected pigs is excessive granulomatous inflammation in the lymphoid tissues, genes with granulocyte/leukocyte infiltration function were depicted separately in Tab. 3. All but three of the genes (*ADM*, *TNFAIP3* and *TIMP1*) showed a predicted increasing effect facilitating granulocyte/leukocyte infiltration. Indeed, functional analysis of differentially-expressed transcripts revealed an enrichment of genes associated with immune cell trafficking, inflammatory responses and immunological disease (Fig. 4, Tab. S1).

**Table 3:** Downstream effect analysis of upregulated genes involved in cell migration and infiltration in MoDCs 1

*ID*	*FC*	*Effect of Gene on Granulocyte/Leucocyte Infiltration*
*IL1B*	6.470	Increase
*CXCL2*	4.660	Increase
*IL8*	4.400	Increase
*TNF*	4.170	Increase
*CCL4*	3.370	Increase
*ADM*	3.270	Decrease
*IL1A*	3.200	Increase
*TNFAIP3*	2.140	Decrease
*NFKBIA*	2.080	Increase
*OSM*	1.860	Increase
*TIMP1*	1.770	Decrease

Even with the large number of genes differentially expressed, only 3 canonical pathways reached significance of *p*<0.05 at 24 h p.i., including *Role of IL-17A in Psoriasis* (*p = 0.0136*), *RAN Signalling* (*p = 0.0178*) and *Role of RIG1-like Receptors in Antiviral Innate Immunity* (*p = 0.0454*; Tab. S1, Tab. S3), an important pathway involved in the host recognition of viral PAMPs. Interestingly, none of the top ranking canonical pathways identified by IPA were similar to those identified at the 1 h time-point.

Functional analysis revealed an enrichment of genes involved in *Cell Death*, with largely increasing effects at both time-points (Fig. 4, Tab. 4). PCV2-induced apoptosis may contribute to limiting the host response [42], this may be particularly important during the acute phase of infection when viral replication takes place [43]. An enrichment of differentially expressed genes with functions in apoptosis, both pro- and anti-apoptotic, were identified in MoDC at both time-points with a shift towards a more pro-apoptotic state at the later time-point where six out of nine genes have a predicted increasing effect (Tab. 4). Further network analyses depicting the top-networks of interacting genes for MoDC 1 h and 24 h after PCV2b infection are shown in figures S3 and S4.

**Table 4:** Downstream effect analysis of differentially-expressed genes involved in apoptosis in MoDCs 1 h and 24 h post PCV2b challenge.

*Cell Type*	*ID*	*FC*	*Predicted Effect of Gene on Apoptosis*
***MoDCs 1 h p.i.***			
	*IL1B*	6.470	Increased
	*IL8*	4.400	Increased
	*TNF*	4.170	Increased
	*IER3*	3.400	Increased
	*IL1A*	3.200	Increased
	*MAP3K8*	2.910	Increased
	*IRF1*	2.100	Increased
	*NFKBIA*	2.080	Increased
	*OSM*	1.860	Increased
	*HBEGF*	1.840	Affected
	*CXCL2*	4.660	Decreased
	*CCL4*	3.370	Decreased
	*ADM*	3.270	Decreased
	*BTG2*	2.350	Decreased
	*RND3*	2.290	Decreased
	*JAG1*	2.150	Decreased
	*TNFAIP3*	2.140	Decreased
	*TIMP1*	1.770	Decreased
	*INSR*	1.600	Decreased
***MoDCs 24 h p.i.***			
	*S100A9*	3.3	Increase
	*SLC25A6*	2.71	Increase
	*RTN4*	2.25	Increase
	*CASP10*	1.78	Increase
	*RANBP1*	1.77	Affects
	*VDAC2*	1.66	Increase
	*BTC*	−1.77	Increase
	*ITGB5*	−2.03	Decrease
	*PIK3IP1*	−2.05	Decrease
	*DKK3*	−2.29	Decrease

### Functional pathway analysis reveals significant activation of cellular development pathways in PCV2b infected BMCs

While no differential gene expression was detected at one hour post infection, 18 genes showed differential expression at 24 h with seven genes significantly upregulated.

**Figure 5:**
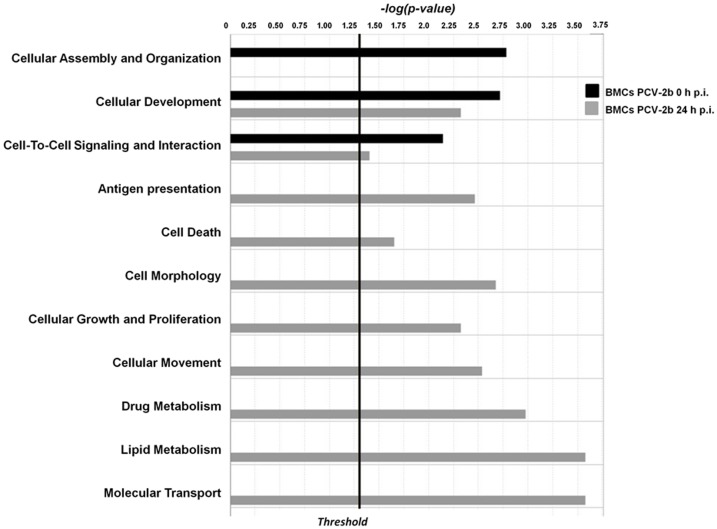
Biological process analysis of genes differentially expressed in BMCs at 1 h and 24 h post PCV2b challenge. Differentially expressed genes (P<0.05) were imported into IPA Ingenuity software to determine significantly enriched biological processes. Data represent the distribution in cell function categories of statistically significantly enriched biological processes (P<0.05) at 0 h and 24 h post PCV2b challenge.

To gain a further insight into the potential mechanisms of the effects of PCV2b challenge on BMCs, the identified transcripts were mapped to networks available in the Ingenuity database. Three networks were identified by scores between three and 13. The scores take the number of focus molecules and the size of the network into account to estimate the relevance of this network. In accordance with the enrichment of genes with tissue development functions (Fig. 5), the highest-scoring network (Network Score 13) revealed a significant link with connective tissue development and function, embryonic and organ development (Fig. S2, Suppl. Fig. 5, Tab. S3).

Among the top ranking canonical pathways identified by IPA, several impact on immune response, including *Eicosanoid Signalling* (1^st^ ranked), important for its contribution to the inflammatory response in a variety of diseases (such as arthritis and asthma) and *CXCR4 Signalling* pathway (9^th^ ranked) (Tab. S3). Prostaglandin E Synthase (*PTGES*; *FC = 2.54*) was central to most pathways and functions affected and has been shown to be induced by IL1- and NFκB during inflammatory conditions [44]. Prostaglandin E Synthase converts prostaglandin endoperoxide H2 (PGH2) to prostaglandin E2 (PGE2), which is a potent immunoregulatory lipid mediator with key roles in the regulation of virus replication and modulation of inflammatory responses following infection [45]. Indeed, PGE2 has not only been shown to downregulate IL-12 and IL-23 production, two of the major driving cytokines for a Th1 response [46], but also leads to the development of myeloid-derived suppressor cells [47] and has dampening effects on MoDC maturation [48].

## Discussion

PCV-2 is the causative agent of PMWS, a widespread complex multifactorial disease with recognized immunosuppressive characteristics [18]. Compelling evidence suggests that the intricate interaction between the host immune system to the presence of PCV2 and the ability of PCV2 to interfere with immune defence is a key event in the pathological outcome of PMWS. While productive replication of PCV-2 is most likely restricted to epithelial and endothelial cells [32], [49], the virus has been shown to infect and persist in cells of the immune system, including MØ, DC and lymphocytes [14], [50]–[52]. The current study assessed the early molecular mechanisms involved in PCV2 infection of immune cell subsets using a genome-wide expression approach. To aid to the clinical significance of this study, a pathogenic PCV2b strain currently circulating in the UK was used.

Similar as described in earlier publication [14], [53], PCV2b seems to readily infection of all three cell types in the absence of clear viral replication. It is also interesting to mention that there does not seem to be a correlation between viral copy numbers in different cell-types and changes in gene expression levels as analysed by microarray. Agilent microarray analysis of the transcriptome differences between challenged and non-challenged cells revealed a reduced host response of AMØs and BMCs to PCV2b-challenge *in vitro* at both early and later time-points. This result was particularly surprising considering AMØs are among the first innate immune cell to encounter PCV2 in the lung [54] and function in phagocytic clearance, inflammatory reactions and tissue homeostasis [55], but may also be partially affected by the experimental set-up in which cells were exposed to PCV2b at 0 h, and virus was washed off again straight away. However, this early immunological ignorance of AMØs towards PCV2b infection has been observed by others [53]. Indeed, a recent study utilising a proteomics approach of AMØs infected with PCV2 revealed no obvious changes at 24 h p.i., but resulted only in 9 up-regulated and 12 down-regulated proteins at 48 h p.i. [56], which included cytoskeleton proteins, macromolecular biosynthesis-associated proteins, stress response proteins, signal transduction proteins, energy metabolism, and ubiquitin proteasome pathway-associated proteins. Moreover, nine corresponding genes of the differentially expressed proteins were quantified by real time RT-PCR to examine the transcriptional profiles. Thus, our data regarding gene-expression differences in AMØs fit very well to these data described by proteome-analysis. A decreased immune recognition and response to PCV2b may be a genuine feature of these cells. During the writing of this manuscript, results of a study were published [57] showing that PCV2 infection of AMØs resulted in differential regulation of far more genes than described here or in Cheng et al [56]. Whereas the differences between these results are not completely clear, they can potentially attributed to using a different mock-infection (supernatant of PK15 cells here versus media) or the MOI and virus strain used (UK strain versus Chinese strain, MOI of 0.5 versus MOI of 1). Indeed, several publications have shown that PCV2 isolates from China show a rearranged genotype [58]–[62]. Further explanation for the differences in results could be attributed to the treatment of cells (frozen-thawed versus fresh in the current study) as well as the testing for LPS (all reagents tested for LPS in the current study), which may also explain the TLR activation described in the recent publication [57]. Thus, whereas both studies identified the same central molecules, the resulting differences may be attributed to how the AMØs were treated rather than virus specific differences.

AMØs (24 h) and MoDCs (1 h) had increased gene expression levels of TNF which also represented the only transcript common to two of the three immune cells. TNF is produced by activated MØs in response to microbial/viral stimuli and regulates a wide range of biological activities, including cell differentiation, proliferation and death, as well as inflammatory responses and innate and adaptive immune responses [63]–[65]. An increase in TNF expression in AMØs and MoDCs in response to viral triggers agrees well with previous *in vivo* studies. Indeed, Kim et al. (2006) [66] showed excessive production of TNFá by porcine parvovirus (PPV) in a dual infection model *in vivo*, while PCV-2-challenge alone did not induce TNFá to the same extent. Similar results were obtained in a PRRSV/PCV-2 dual infection model of AMØs *in vitro* [67], [68]. Interestingly, in this model the excessive production of TNF by the co-infecting pathogen potentiated PCV-2-induced PMWS. Signalling through TNF activates various downstream cascades, including activation of transcription factor NF-κB and pro-apoptotic pro-caspases 8 and 10 [63]. In support of this is the enrichment of genes in the *NF-κB Signalling* pathway in MoDCs 1 h post PCV2b-challenge (Suppl. Tab. 1), the up-regulation of caspase 10 and an enrichment of genes with mainly pro-apoptotic functions 24 h post-challenge. Thereby, our findings further demonstrate a clear role for TNF in the response to PCV-2 challenge in AMØs and MoDCs, substantiating a role of this key cytokine in the aetiology of PMWS.

Further functional analysis of MoDCs revealed an enrichment of genes involved in apoptosis with largely increasing effects at both time-points (Tab. 4). The role of apoptosis in the development of PMWS remains under investigation. While T- and B-lymphocyte depletion in lymphoid tissues, lymphopenia in peripheral blood and a reduction of B cells is a hallmark in pigs that progress to develop clinical signs of PMWS [69], this decline may similarly result from reduced proliferation rather than increased apoptosis [43]. However, it has been shown that ORF3 facilitates PCV-2-induced apoptosis *in vitro* and *in vivo* [70]. This study has identified an increased expression of more than 15 pro-apoptotic genes at both time-points suggesting a potential role for apoptosis in the pathogenesis of PMWS and characteristic lesions.

Significant upregulation of five genes within the ‘*Proinflammatory Hypercytokinemia/hyperchemokinemia*’ pathway (Fig. S1) indicated a strong pro-inflammatory response associated with PCV2b challenge in MoDCs, which resemble inflammatory DC *in vivo* [71], leading to the recruitment of immune cells to the site of infection. Indeed, a unique histopathological characteristic of PMWS-affected pigs is excessive granulomatous inflammation in lymphoid and other tissues [72]. Pathogenesis of these immune mediated lesions includes the recruitment of circulating monocytes to the site of inflammation [73]. This is facilitated by signalling through NFκB, p38, or MAPKs-mediated regulation of pro-inflammatory cytokine expression (IL1-β, TNF and IL-6) together with chemokines and cell adhesion proteins [74]. This was further supported by the enrichment of differentially-expressed transcripts with biological functions in immune cell trafficking and cell-to-cell signalling and the PCV2b-induced stimulation of pro-inflammatory cytokines and chemoattractants as seen in MoDC are similar to those seen in subclinically PCV2 infected pigs [25].

Antiviral immune responses *in vivo* are mediated by a variety of cell types, thus, it is important to understand the dynamic interaction between the immune cell subsets and viral pathogens, including PCV2. Our present study has described the transcriptional response of three immune cell subsets challenged with the dominant UK PCV2b strain currently circulating on severely PMWS-affected farms.

Analysis of the differentially-expressed genes showed a distinct cell-type dependent response to PCV2b challenge and identified several key molecules, functions and pathways involved in the host response to PCV2b. The better understanding of these mechanisms could provide new approaches to PCV2 infection and the development of novel immunotherapies or vaccines for the treatment of PMWS.

## Supporting Information

Figure S1 **Pathway ‘Role of Hypercytokinemia/hyperchemokinemia in the Pathogenesis of Influenza’.** Pathway analysis with the IPA software allowed identification of pathways that were differentially expressed between PCV-2b-challenged and unchallenged MoDCs 1 h post infection. The *‘Role of Hypercytokinemia/hyperchemokinemia in the Pathogenesis of Influenza’* pathway was one of the most affected pathways (*P = 5.21E-09*), with five out of the 44 genes present significantly affected. Significant upregulation of five genes within this pathway indicated a strong pro-inflammatory response associated with PCV-2b challenge in MoDCs.
(TIF)

Figure S2 **Network Analysis of MoDC 1 h p.i.** Analysis with the IPA software identified transcripts that could be mapped to networks available in the Ingenuity database. In MoDC 1 h p.i., four networks were identified with the highest ranking network revealing a significant link with Cell-To-Cell Signaling and Interaction, Cellular Growth and Proliferation, Renal and Urological System Development and Function.
(TIF)

Figure S3 **Network Analysis of MoDC 24 h p.i.** Analysis with the IPA software identified transcripts that could be mapped to networks available in the Ingenuity database. In MoDC 24 h p.i., only two networks were identified with the highest ranking network revealing a significant link with Cell Death and Survival, Cellular Development, Hematological System Development and Function.
(TIF)

Figure S4 **Network Analysis of BMCs 24 h p.i.** Analysis with the IPA software identified transcripts that could be mapped to networks available in the Ingenuity database. In BMCs 24 h p.i., the highest ranking network revealed a significant link with Cellular Development, Hematological System Development and Function, Hematopoiesis.
(TIF)

Table S1 **Pathway analysis of genes differentially expressed in between PCV-2b-challenged and non-challenged immune cell subsets.** Differentially expressed genes (P<0.05) were imported into the Ingenuity Pathways Analysis (IPA) software. Canonical pathways analysis identified the pathways from the IPA library of canonical pathways that were most significant to the data set. Molecules from the data set that met the −1.5≤ fold change ≥1.5 and a FDR-adjusted P<0.05 cutoff and were associated with a canonical pathway in the Ingenuity Knowledge Base were considered for the analysis.
(DOCX)

Table S2 **Gene list of DE genes grouped by IPA based on gene function in MoDCs after 1 h and 24 h p.i.** Genes in red and bold represent significantly upregulated transcripts expressed in this study. Genes in green and bold represent significantly downregulated transcripts expressed in this study. A network score of >2 was considered significant (p<0.01).
(DOCX)

Table S3 **Gene list of DE genes grouped by IPA based on gene function in BMCs after 24 h p.i.** Genes in green and bold represent significantly downregulated transcripts expressed in this study. Genes in red and bold represent significantly upregulated transcripts expressed in this study. A network score of >2 was considered significant (p<0.01).
(DOCX)

Table S4 **Pathway analysis of genes differentially expressed in between PCV2b-challenged and non-challenged immune cell subsets.** Differentially expressed genes (P<0.05) were imported into the Ingenuity Pathways Analysis (IPA) software. Canonical pathways analysis identified the pathways from the IPA library of canonical pathways that were most significant to the data set. Molecules from the data set that met the −1.5≤ fold change ≥1.5 and a FDR-adjusted P<0.05 cutoff and were associated with a canonical pathway in the Ingenuity Knowledge Base were considered for the analysis.
(DOCX)
