# Supplementary figures and images for: Global Gene Expression Profiling of Myeloid Immune Cell Subsets in Response to In Vitro Challenge with Porcine Circovirus 2b
Source: PLoS One. 2014 Mar 11;9(3):e91081. doi: 10.1371/journal.pone.0091081 (PMC3949749; doi:10.1371/journal.pone.0091081)

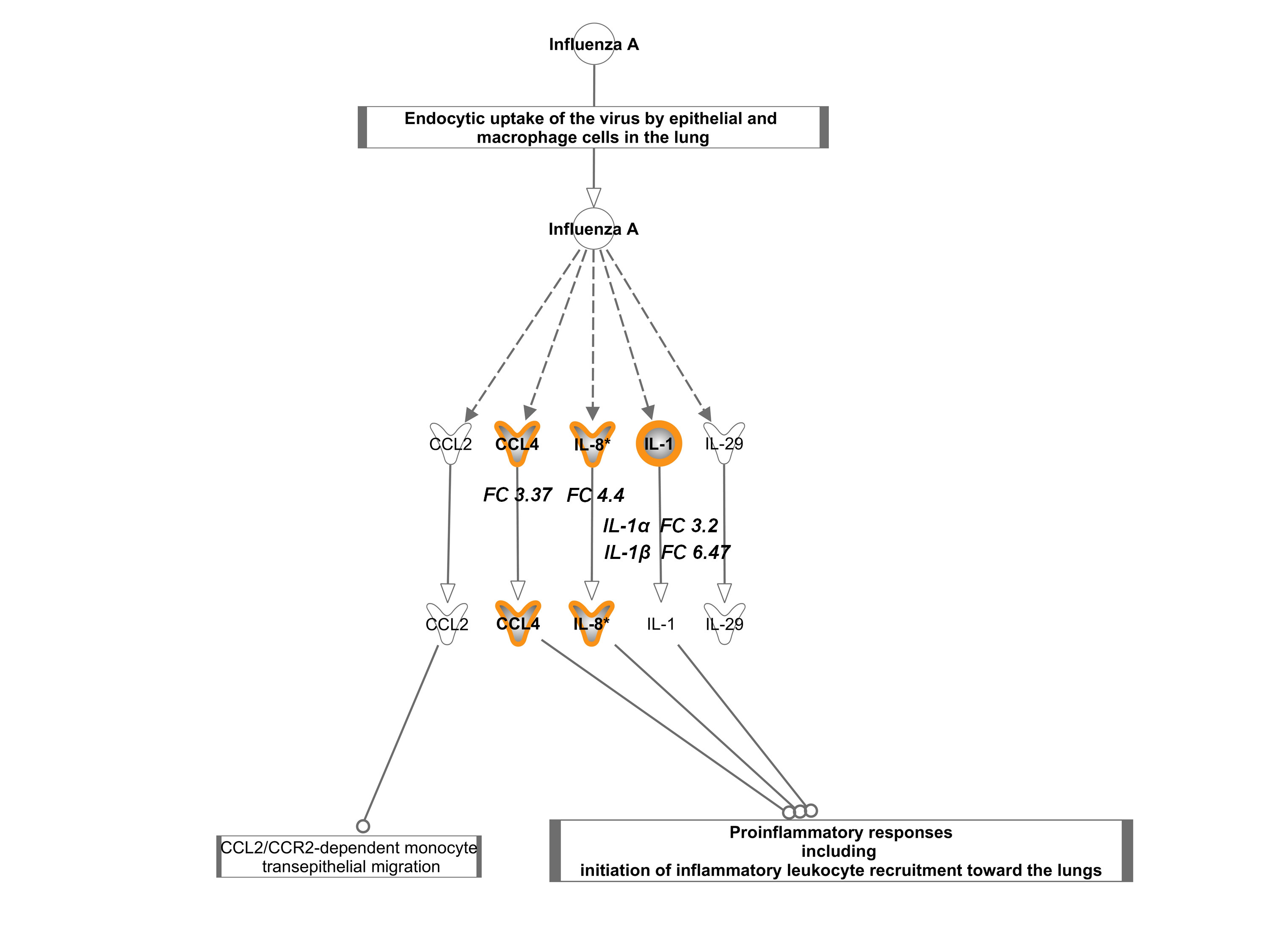

Supplement: Figure S1 — Pathway ‘Role of Hypercytokinemia/hyperchemokinemia in the Pathogenesis of Influenza’. Pathway analysis with the IPA software allowed identification of pathways that were differentially expressed between PCV-2b-challenged and unchallenged MoDCs 1 h post infection. The ‘Role of Hypercytokinemia/hyperchemokinemia in the Pathogenesis of Influenza’ pathway was one of the most affected pathways (P = 5.21E-09), with five out of the 44 genes present significantly affected. Significant upregulation of five genes within this pathway indicated a strong pro-inflammatory response associated with PCV-2b challenge in MoDCs. (TIF) [file pone.0091081.s001.tif]

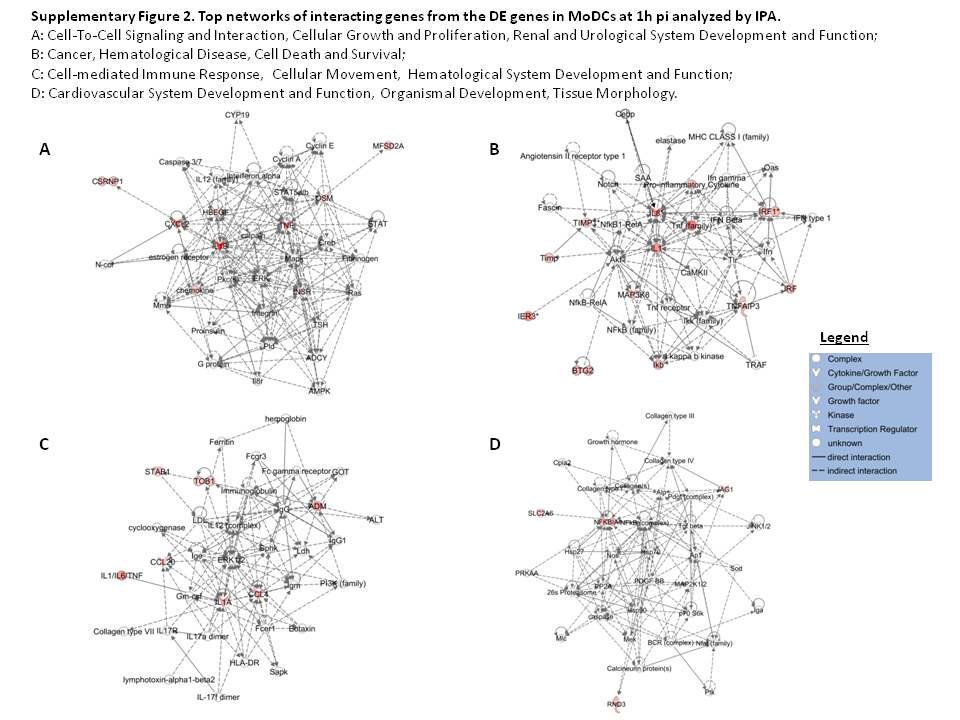

Supplement: Figure S2 — Network Analysis of MoDC 1 h p.i. Analysis with the IPA software identified transcripts that could be mapped to networks available in the Ingenuity database. In MoDC 1 h p.i., four networks were identified with the highest ranking network revealing a significant link with Cell-To-Cell Signaling and Interaction, Cellular Growth and Proliferation, Renal and Urological System Development and Function. (TIF) [file pone.0091081.s002.tif]

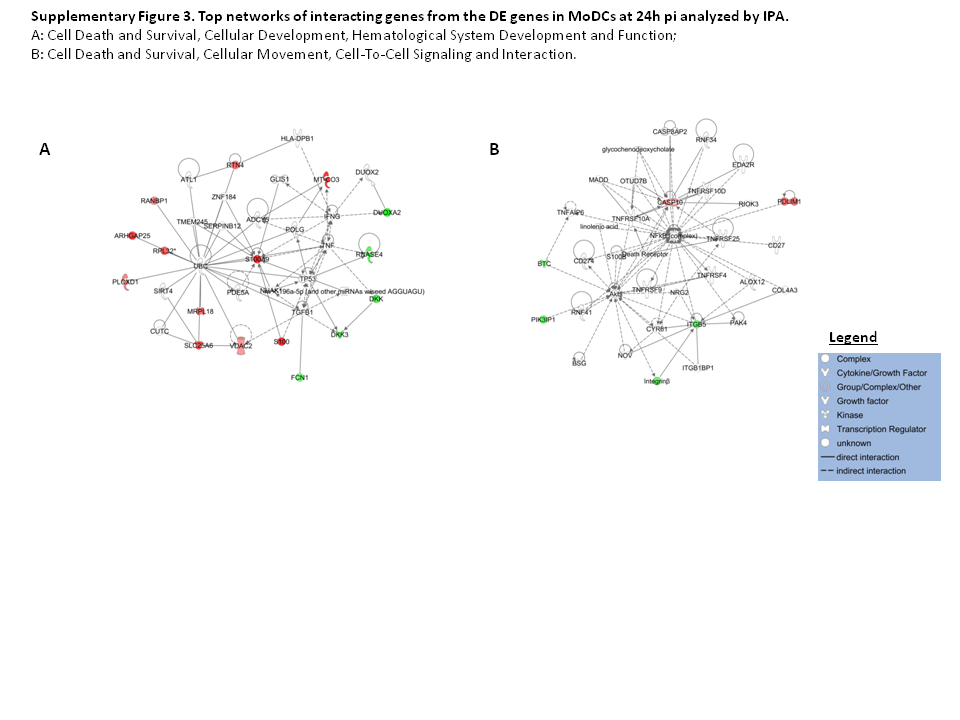

Supplement: Figure S3 — Network Analysis of MoDC 24 h p.i. Analysis with the IPA software identified transcripts that could be mapped to networks available in the Ingenuity database. In MoDC 24 h p.i., only two networks were identified with the highest ranking network revealing a significant link with Cell Death and Survival, Cellular Development, Hematological System Development and Function. (TIF) [file pone.0091081.s003.tif]

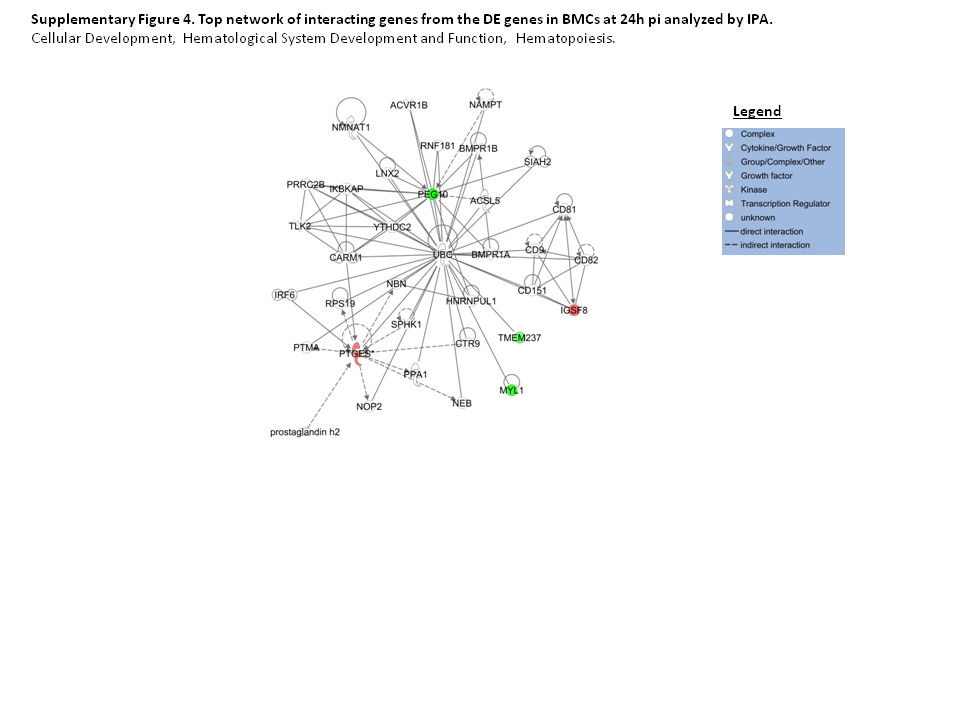

Supplement: Figure S4 — Network Analysis of BMCs 24 h p.i. Analysis with the IPA software identified transcripts that could be mapped to networks available in the Ingenuity database. In BMCs 24 h p.i., the highest ranking network revealed a significant link with Cellular Development, Hematological System Development and Function, Hematopoiesis. (TIF) [file pone.0091081.s004.tif]
