# Supplementary material for: Global Gene Expression Profiling of Myeloid Immune Cell Subsets in Response to In Vitro Challenge with Porcine Circovirus 2b
Source: PLoS One. 2014 Mar 11;9(3):e91081. doi: 10.1371/journal.pone.0091081 (PMC3949749; doi:10.1371/journal.pone.0091081)
Supplement: Table S2 — Gene list of DE genes grouped by IPA based on gene function in MoDCs after 1 h and 24 h p.i. Genes in red and bold represent significantly upregulated transcripts expressed in this study. Genes in green and bold represent significantly downregulated transcripts expressed in this study. A network score of >2 was considered significant (p<0.01). (DOCX) [file pone.0091081.s006.docx]

**Table S2: Gene list of DE genes grouped by IPA based on gene function in MoDCs after 1h and 24h p.i.**

Genes in red and bold represent significantly upregulated transcripts expressed in this study. Genes in green and bold represent significantly downregulated transcripts expressed in this study. A network score of ＞2 was considered significant (p＜0.01)

| **Cell Type** | **ID** | **Molecules in network (1 HPI)** | **Score** | **Focus Molecules** | **Top Functions** |
| --- | --- | --- | --- | --- | --- |
| **MoDCs** | 1 | ADCY, AMPK, calpain, Caspase 3/7, chemokine, Creb, **CSRNP1**, **CXCL2**, Cyclin A, Cyclin E, CYP19, ERK, estrogen receptor, Fibrinogen, G protein, **HBEGF**, IL12 (family), **IL1B**, Il8r, **INSR**, Integrin, Interferon alpha, Mapk, **MFSD2A**, Mmp, N-cor, **OSM**, Pkc(s), Pld, Proinsulin, Ras, STAT, STAT5a/b, **TNF**, TSH | 17 | 8 | Cell-To-Cell Signaling and Interaction, Cellular Growth and Proliferation, Renal and Urological System Development and Function |
|  | 2 | Akt, Angiotensin II receptor type 1, **BTG2**, CaMKII, Cebp, elastase, Fascin, I kappa b kinase, **IER3**, Ifn, IFN Beta, Ifn gamma, IFN type 1, Ikb, Ikk (family), IL1, **IL8**, **IRF1**, IRF, **MAP3K8**, MHC CLASS I (family), NFkB (family), NfkB-RelA, NfkB1-RelA, Notch, Oas, Pro-inflammatory Cytokine, SAA, **TIMP1**, Timp, Tlr, Tnf (family), Tnf receptor, **TNFAIP3**, TRAF | 15 | 7 | Cancer, Hematological Disease, Cell Death and Survival |
|  | 3 | **ADM**, ALT, **CCL4**, **CCL20**, Collagen type VII, cyclooxygenase, Eotaxin, ERK1/2, Fc gamma receptor, Fcer1, Fcgr3, Ferritin, Gm-csf, GOT, hemoglobin, HLA-DR, Ige, IgG1, IgG, Igm, IL-17f dimer, IL1/IL6/TNF, IL12 (complex), IL17a dimer, IL17R, **IL1A**, Immunoglobulin, Ldh, LDL, lymphotoxin-alpha1-beta2, PI3K (family), Sapk, Sphk, **STAB1**, **TOB1** | 12 | 6 | Cell-mediated Immune Response, Cellular Movement, Hematological System Development and Function |
|  | 4 | 26s Proteasome, Alp, Ap1, BCR (complex), Calcineurin protein(s), caspase, Collagen type I, Collagen type III, Collagen type IV, Collagen(s), Cpla2, Growth hormone, Hsp27, Hsp70, Hsp90, Iga, **JAG1**, JINK1/2, MAP2K1/2, Mek, Mlc, Nfat (family), NFkB (complex), **NFKBIA**, Nos, p70 S6k, Pdgf (complex), PDGF BB, PP2A, PRKAA, Ptk, **RND3**, **SLC2A6**, Sod, Tgf beta | 7 | 4 | Cardiovascular System Development and Function, Organismal Development, Tissue Morphology |
|  |  | **Molecules in network (24 HPI)** | **Score** | **Focus Molecules** | **Top Functions** |
|  | 1 | ADCY5, **ARHGAP25**, ATL1, CUTC, **DKK3**, DKK, DUOX2, **DUOXA2**, **FCN1**, GLIS1, HLA-DPB1, IFNG, miR-196a-5p (and other miRNAs w/seed AGGUAGU), **MRPL18**, **MT-CO3**, NUAK1, PDE5A, **PLCXD1**, POLG, **RANBP1**, **RNASE4**, **RPL32**, **RTN4**, S100, **S100A9**, SERPINB12, SIRT4, **SLC25A6**, TGFB1, TMEM245, TNF, TP53, UBC, **VDAC2**, ZNF184 | 37 | 14 | Cell Death and Survival, Cellular Development, Hematological System Development and Function |
|  | 2 | Akt, ALOX12, BSG, **BTC**, **CASP10**, CASP8AP2, CD27, CD274, COL4A3, CYR61, Death Receptor, EDA2R, glycochenodeoxycholate, Integrinβ, **ITGB5**, ITGB1BP1, linolenic acid, MADD, NFkB complex), NOV, NRG2, OTUD7B, PAK4, **PDLIM1**, **PIK3IP1**, RIOK3, RNF34, RNF41, S100B, TNFAIP6, TNFRSF4, TNFRSF9, TNFRSF25, TNFRSF10A, TNFRSF10D | 11 | 5 | Cell Death and Survival, Cellular Movement, Cell-To-Cell Signaling and Interaction |
