# Supplementary material for: Global Gene Expression Profiling of Myeloid Immune Cell Subsets in Response to In Vitro Challenge with Porcine Circovirus 2b
Source: PLoS One. 2014 Mar 11;9(3):e91081. doi: 10.1371/journal.pone.0091081 (PMC3949749; doi:10.1371/journal.pone.0091081)
Supplement: Table S3 — Gene list of DE genes grouped by IPA based on gene function in BMCs after 24 h p.i. Genes in green and bold represent significantly downregulated transcripts expressed in this study. Genes in red and bold represent significantly upregulated transcripts expressed in this study. A network score of >2 was considered significant (p<0.01). (DOCX) [file pone.0091081.s007.docx]

| **Table S3: Gene list of DE genes grouped by IPA based on gene function in BMCs after 24h pi.** | | | | | |
| --- | --- | --- | --- | --- | --- |
| Genes in green and bold represent significantly downregulated transcripts expressed in this study. Genes in red and bold represent significantly upregulated transcripts expressed in this study. A network score of ＞2 was considered significant (p＜0.01). | | | | | |
| **Cell Type** | **ID** | **Molecules in network (1 HPI)** | **Score** | **Focus Molecules** | **Top Functions** |
| **BMCs** | 1 | ACSL5, ACVR1B, BMPR1A, BMPR1B, CARM1, CD9, CD81, CD82, CD151, CTR9, HNRNPUL1, **IGSF8**, IKBKAP, IRF6, LNX2, **MYL1**, NAMPT, NBN, NEB, NMNAT1, NOP2, **PEG10**, PPA1, prostaglandin h2, PRRC2B, **PTGES**, PTMA, RNF181, RPS19, SIAH2, SPHK1, TLK2, **TMEM237**, UBC, YTHDC2 | 13 | 5 | Cellular Development, Hematological System Development and Function, Hematopoiesis |
|  | 2 | **MMRN2**, VDR | 3 | 1 | Cardiovascular Disease, Cell Morphology, Cellular Development |
|  | 3 | arylesterase, **Es25** | 3 | 1 |  |
