# Supplementary material for: Global Gene Expression Profiling of Myeloid Immune Cell Subsets in Response to In Vitro Challenge with Porcine Circovirus 2b
Source: PLoS One. 2014 Mar 11;9(3):e91081. doi: 10.1371/journal.pone.0091081 (PMC3949749; doi:10.1371/journal.pone.0091081)
Supplement: Table S4 — Pathway analysis of genes differentially expressed in between PCV2b-challenged and non-challenged immune cell subsets. Differentially expressed genes (P<0.05) were imported into the Ingenuity Pathways Analysis (IPA) software. Canonical pathways analysis identified the pathways from the IPA library of canonical pathways that were most significant to the data set. Molecules from the data set that met the −1.5≤ fold change ≥1.5 and a FDR-adjusted P<0.05 cutoff and were associated with a canonical pathway in the Ingenuity Knowledge Base were considered for the analysis. (DOCX) [file pone.0091081.s008.docx]

***Table S4: Pathway analysis of genes differentially expressed in between PCV2b-challenged and non-challenged immune cell subsets.***

Differentially expressed genes (P<0.05) were imported into the Ingenuity Pathways Analysis (IPA) software. Canonical pathways analysis identified the pathways from the IPA library of canonical pathways that were most significant to the data set. Molecules from the data set that met the -1.5 ≤ fold change ≥ 1.5 and a FDR-adjusted P<0.05 cutoff and were associated with a canonical pathway in the Ingenuity Knowledge Base were considered for the analysis.

| **Pathway** | **P-value** | **Sign.*** | **Total**** |
| --- | --- | --- | --- |
| **AMΦs 0 h p.i.** | | | |
| Methane Metabolism | 1.12E-03 | 1 | 67 |
| Stilbene, Coumarine and Lignin Biosynthesis | 1.23E-03 | 1 | 75 |
| Phenylalanine Metabolism | 2.50E-03 | 1 | 111 |
| **AMΦs 1 h p.i.** | | | |
| Airway Inflammation in Asthma | 2.63E-04 | 1 | 6 |
| Airway Pathology in Chronic Obstructive Pulmonary Disease | 5.26E-04 | 1 | 9 |
| Differential Regulation of Cytokine Production in Macrophages and T Helper Cells by IL-17A & IL-17F | 1.18E-03 | 1 | 18 |
| **MoDCs 1 h p.i.** | | | |
| NF-kB Signaling | 3.61E-09 | 7 | 175 |
| Role of Hypercytokinemia/hyperchemokinemia in the Pathogenesis of Influenza | 5.21E-09 | 5 | 44 |
| Communication between Innate and Adaptive Immune Cells | 2.25E-07 | 5 | 110 |
| PPAR Signaling | 2.64E-07 | 5 | 106 |
| IL-6 Signaling | 7.16E-07 | 5 | 124 |
| Role of Cytokines in Mediating Communication between Immune Cells | 1.29E-06 | 4 | 55 |
| IL-10 Signaling | 3.04E-06 | 4 | 78 |
| **MoDCs 24 h p.i.** | | | |
| Role of IL-17A in Psoriais | 0.0136 | 1 | 13 |
| RAN Signalling | 0.0178 | 1 | 24 |
| Role of RIG1-like Receptors in Antiviral Innate Immunity | 0.0454 | 1 | 49 |
| **BMCs 24 h p.i.** | | | |
| Eicosanoid Signaling | 0.0157 | 1 | 79 |
| Regulation of Actin-based Motility by Rho | 0.0219 | 1 | 91 |
| PAK Signaling | 0.0232 | 1 | 107 |

|  |
| --- |
| * The number of genes in each pathway that are differentially expressed |
| ** Total number of genes associated with pathway |
